# A Mitomycin C-Sparing Novel Technique for Subscleral Trabeculectomy in Primary Congenital Glaucoma

**DOI:** 10.1155/2020/2017158

**Published:** 2020-06-07

**Authors:** Ashraf Bor'i, Salah M. Al-Mosallamy, Tamer G. Elsayed, Wael M. El-Haig

**Affiliations:** Ophthalmology Department, Faculty of Medicine, Zagazig University, Zagazig, Egypt

## Abstract

**Purpose:**

To evaluate the safety and efficacy of a novel modified subscleral trabeculectomy technique in management of primary congenital glaucoma.

**Methods:**

This study included 25 infants diagnosed of having bilateral primary congenital glaucoma. For each patient, one eye was assigned to undergo subscleral trabeculectomy with trimming of the edges of the scleral bed (group I), while the contralateral eye underwent subscleral trabeculectomy with application of mitomycin C (0.4 mg/ml for 3 min) (group II). All the patients were followed up for a period of 14 ± 3 months (range 13–22 months).

**Results:**

25 eyes were included in each group. Patients' mean age was 2.5 ± 0.5 months (range 1.8–6.5 months). The mean preoperative intraocular pressure was 31 ± 4.9 mmHg and 32.1 ± 4.0 mmHg in group I and II, respectively. The mean postoperative intraocular pressure was 9.0 ± 1.0, 11.0 ± 3.2, 12.5 ± 0.9, 13.0 ± 2.9, and 15.5 ± 1.5 mm Hg in group I and was 10.3 ± 1.2, 12.0 ± 2.5, 13.5 ± 1.7, 15.0 ± 1.5, and 17.1 ± 2.8 mm Hg in group II at the first week and 1, 3, 6, and 12 months, respectively. There was no statistically significant difference between the mean intraocular pressure values recorded at both groups preoperatively and at each follow-up visit. Failure necessitating further surgical interventions was recorded in 4 eyes (16%) in group I as compared to 3 eyes (12%) in group II (*P* > 0.05). Postoperative complications included mild hyphema, which occurred in one eye (4%) in group I and 2 eyes (8%) in group II, and shallow anterior chamber in 3 eyes (12%) in group I and in 2 eyes (8%) in group II. One eye (4%) in group I developed drawn-up pupil. Choroidal effusion developed in one eye (4%) at each group.

**Conclusion:**

Trimming the edges of the scleral bed adjacent to the sclera flap is a safe and effective surgical step which can be added to the subscleral trabeculectomy procedure to effectively control the intraocular pressure in patients with primary congenital glaucoma, sparing them the hazards associated with mitomycin C application.

## 1. Introduction

Primary congenital glaucoma (PCG) represents diagnostic and therapeutic challenges to ophthalmologists and may lead to visual handicap [1].

Subscleral trabeculectomy entails the induction of a new pathway for aqueous permeation between the anterior chamber (AC) and the subconjunctival space, beneath the scleral flap. Accordingly, after iridectomy and sclerectomy, the underside of the partial thickness scleral flap, the sides of the scleral wound, and the area between the episclera and conjunctiva must remain free from healing or scarring to prevent obstruction of the aqueous outflow through the fistula [2].

In congenital glaucoma, trabeculectomy shows a wide range of success (35% to 80%). This variability in outcome may be attributed to different factors like the patient age, associated ocular or systemic abnormalities, the use of adjuvant antimetabolites, and the variable duration of follow-up between different studies [3–5]. Halting or delaying wound healing in the early postoperative period allows steady egress of aqueous through the newly fashioned pathway and may improve the results [6].

Intraoperative modulation of wound healing, which starts just before creating the scleral wound, is of extreme significance in reducing postoperative scarring. Although meticulous surgery and good tissue handling with intraoperative complete hemostasis are mandatory to minimize both coagulative and inflammatory phases of wound healing, they are usually ineffective for the prevention of scar formation. Therefore, intraoperative healing modulators including anti-inflammatory agents, antifibrotics, antivascular endothelial growth factors (anti-VEGFs), and physical spacers have been used to achieve better control on wound healing [2, 7, 8]. Mitomycin C (MMC) is a potent antifibrotic agent that can prevent or decrease fibroblastic activity, hence reducing fibrous tissue formation at the fistula site that may improve bleb survival with the resultant better control of the intraocular pressure (IOP) [9]. Owing to the prolonged inhibitory effect of MMC on fibroblast proliferation (about one month) and its nonspecific action on cell biology and metabolism, this improved success has been associated with a higher incidence of both early postoperative complications such as hypotony, shallow AC, early wound leaks, conjunctival necrosis, and choroidal effusions and late postoperative complications such as hypotony-related maculopathy, excessively thin-walled blebs, corneal epitheliotoxicity, late-onset bleb leaks, and bleb-related endophthalmitis [10].

The current study was conducted to evaluate the safety and efficacy of a new modified design for subscleral trabeculectomy technique, aiming to control the postoperative IOP without using MMC in patients with PCG.

## 2. Patients and Methods

Twenty five Egyptian infants diagnosed with bilateral PCG, suspected clinically with photophobia, large corneal diameter, or lacrimation, and proved on examination under general anesthesia were included in the study. All participants had an informed consent form signed by their natural guardians. The protocol for the work has been approved by the Ethics Committee of the institution within which the work was carried out, and it conforms to the provisions of the Declaration of Helsinki in 1995 (as revised in Edinburgh 2000).

Preoperative evaluation included careful history taking obtained from parents. Ocular examination was done under general anesthesia (nitrous oxide and halothane with oxygen) before surgery; AC depth and corneal clarity were examined under high magnification using a hand-held slit lamp (Zeiss HSO 10 Hand slit lamp) to exclude the presence of any associated corneal or iris abnormalities and detect corneal clarity. Gonioscopy was done in all eyes, measurement of IOP was done by the Perkins hand-held applanation tonometer, and horizontal corneal diameter was measured by a caliper. Fundus examination was done using direct and indirect ophthalmoscopy to detect cupping, and topical glycerine solution was used to improve visualization. Axial length measurement was done using A-scan biometry.

Infants with proved PCG received beta blockers eye drops twice daily till the time of surgery. The patients were randomly (using random computer-generated numbers) allocated to receive a modified subscleral trabeculectomy, in which trimming of the edges of the sclera bed was carried out after suturing the superficial scleral flap (group I). The contralateral eye was managed by subscleral trabeculectomy with intraoperative application of MMC (group II) and served as a control group. At least one week lag was respected between the surgery of the first eye and the fellow eye of each patient.

### 2.1. Surgical Technique

Subscleral trabeculectomy was performed under general anesthesia in all patients in both groups. A superior fornix-based conjunctival flap was created by dissecting the conjunctiva and Tenon's capsule from the limbus over an area of approximately 5 mm. After cauterization and hemostasis, about one-half of partial thickness quadrangular scleral flap 3 × 3 mm was dissected toward the clear cornea.

#### 2.1.1. Group I

**Figure 1:**
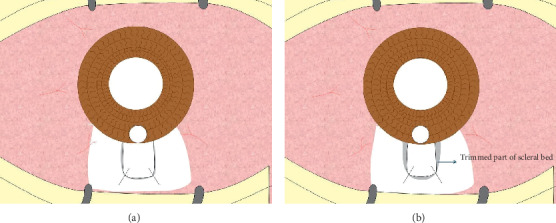
Closure of the flap with 2 nylon sutures (a) and the trimmed 3 edges of the scleral bed (b).

After a superotemporal paracentesis was done, 2 × 2 mm trabeculectomy was excised followed by peripheral iridectomy. The scleral flap was secured to its bed by 2 water-tight 10-0 nylon sutures. The three edges of the scleral bed adjacent to the sclera flap were trimmed using the fine Vannas scissors, enhancing the potential space for aqueous outflow. Partial excision of Tenon's capsule was performed at the site overlying the scleral flap using a toothed forceps to grasp it and pulling it centrally and avoiding injury to the conjunctiva. Finally, closure of the conjunctiva was done using inverted 10-0 nylon sutures (Figure 1).

#### 2.1.2. Group II

Intraoperative MMC was used. A solution of 0.4 mg/ml MMC (Mitomycin C Kyowa, Biochem Pharmaceutical Inc., Mumbai, India) was prepared, and a 2 × 4 mm surgical sponge soaked in the MMC solution was held in contact under the scleral flap and extended to the area under the conjunctiva and Tenon. The conjunctiva was stretched over the sponge and held in place for 2 min. Subsequently, after the removal of the sponge, careful irrigation of the area that was exposed to MMC with copious amount of saline solution was done such that the falling fluid did not come in contact with the cornea. Paracentesis, trabeculectomy, and peripheral iridectomy were done followed by scleral flap, paracentesis, tenonectomy, and conjunctival closure as in group I.

Postoperatively, all patients received intensive steroids, antibiotics, and cycloplegic eye drops daily. The antibiotic preparation was stopped at two weeks postoperatively, topical cycloplegics were given twice daily and continued for three weeks postoperatively, and the steroid eye drops were instilled every two hours initially to be tapered gradually over six weeks.

### 2.2. Data Collection and Follow-Up

Patients' demographics, horizontal corneal diameter, preoperative IOP, CD ratio, and axial length were collected prior to surgical interventions. Patients were followed up at 1 week, 1 month, 3 month, 6 month, and 12 month postoperatively. Ophthalmic examination was done under general anesthesia to measure the IOP and detect any postoperative complications. Absolute success was defined as 5 < IOP ≤ 21 mmHg without any additional tension-lowering agents or resurgery and any sight-threatening complications or increase in CD ratio, axial length, or horizontal corneal diameter. Qualified success was defined as the need for the addition of antiglaucoma topical therapy to reach the IOP of more than 5 mmHg and equal to or less than 21 mmHg. At the last follow-up visit, bleb morphology was recorded according to the Indiana Bleb Appearance Grading Scale (IBAGS) including bleb height, extent, vascularity, and leak [11].

### 2.3. Statistical Analysis

Statistical analysis was carried out using SPSS statistical software package (ver. 18.0; SPSS, Inc., Chicago, IL, USA). Student's *t*-test was used to compare between means, and Fisher's exact test or the chi-squared test was used to compare the percentage values. A *P* value less than 0.05 was considered significant.

## 3. Results

This prospective study included 25 patients with PCG. The mean age was 2.5 ± 0.5 months (range 1.8–6.5 months). Fifteen patients (60%) were male, and 10 patients (40%) were female.

Preoperatively, 5 eyes (20%) in each group showed corneal edema without corneal scarring on examination. Postoperatively, corneal edema was only seen in one eye (4%) in each group.

The mean preoperative C/D ratio was (55.9 ± 1.8) and (57.5 ± 2.1) in group I and group II, respectively, and the mean postoperative C/D ratio at the last follow-up visit was (56.2 ± 1.3) and (56.9 ± 1.9) in group I and group II, respectively. These differences were statistically insignificant (*P*=0.15).

The mean preoperative axial length was (20.3 ± 1.7) and (20.5 ± 1.1) in group I and group II, respectively, and the mean postoperative axial length at the last follow-up visit was (21.1 ± 0.8) and (20.9 ± 1.4) in group I and group II, respectively. These differences were statistically insignificant (*P*=0.09).

There was no statistically significant difference between groups I and II with regard to the preoperative mean corneal diameter and IOP. The preoperative corneal diameter was 12.5 ± 1.5 mm in group I and 13 ± 1.2 mm in group II (*P* value = 0.2). The mean preoperative IOP in group I was 31.6 ± 4.9 mmHg and 32.1 ± 4.1 mmHg in group II (*P* value = 0.69) (Table 1).

**Figure 2:**
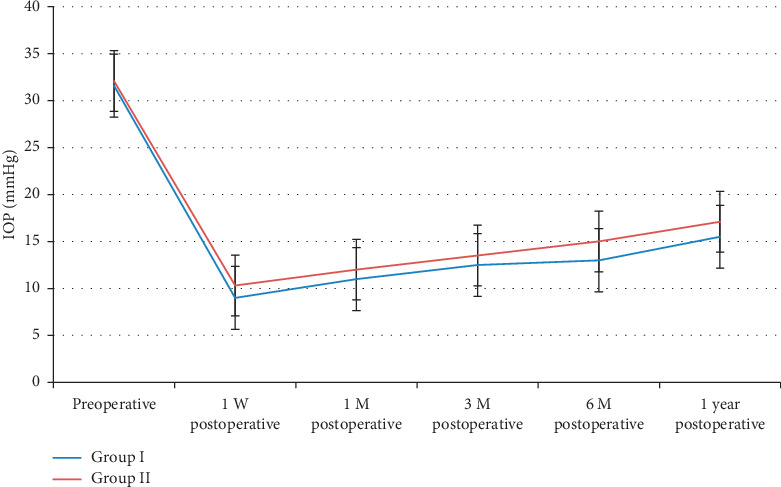
Changes of the mean IOP along the follow-up period.

The mean postoperative IOP was 9.0 ± 1.0, 11.0 ± 3.2, 12.5 ± 0.9, 13.0 ± 2.9, and 15.5 ± 1.50 mm Hg in group I and was 10.3 ± 1.2, 12.0 ± 2.5, 13.5 ± 1.7, 15.0 ± 1.5, and 17.1 ± 2.8 mm Hg in group II at the first week and 1, 3, 6, and 12 months, respectively. These differences were statistically insignificant at each follow-up visit (*P* value = 0.21) (Figure 2).

In group I, 19 eyes (76%) showed absolute success, 2 eyes (8%) showed qualified success, and 4 eyes (16%) showed high IOP and considered as failure and required further surgical intervention to control the IOP. In group II, 17 eyes (68%) showed absolute success, 5 eyes (20%) showed qualified success, and 3 eyes (12%) showed failure and required further surgical intervention to control the IOP. This difference was statistically insignificant between both groups (Table 2).

Intraoperative hyphema occurred in one eye (4%) in group I and in 2 eyes (8%) in group II. Hyphema was mild in all eyes and resolved spontaneously during the first postoperative week.

Early postoperative shallow AC was seen in the first postoperative day which occurred in 3 eyes (12%) in group I and in 2 eyes (8%) in group II; 3 eyes of those with shallow AC resolved spontaneously during the first postoperative week, and only one eye in each group (4%) developed choroidal effusion that resolved completely after reformation of the AC.

Late postoperative drawn-up pupil occurred in one eye in group I (4%) which necessitated no interference as the IOP was controlled, and it did not interfere with the normal pathway of light through the center of the optical media of the eye, thus did not disturb vision. Needling or suture lysis was not needed in any eye during the follow-up period (Table 3).

Comparison of the bleb morphology at the last follow-up visit using the IBAGS (Table 4) revealed insignificant differences between group I and group II with regard to height, extent, vascularity, or aqueous leak.

## 4. Discussion

Although PCG is a rare disease (1 : 10,000 births), it is considered a major cause of visual disability in childhood. The incidence of PCG is 10 times higher in developing countries where consanguineous marriage is common, and these cases may manifest at an earlier age [12, 13].

As the mainstay treatment of pediatric glaucoma is surgical, the long-term control of the IOP is crucial. Goniotomy, trabeculotomy, trabeculectomy, or combinations of these surgical procedures were proposed for treatment of PCG with variability in the rates of success [12].

Trabeculectomy is the preferred surgical technique for some surgeons for some good reasons: when there is no or less familiarity with angle surgery, angle surgery has failed, or the presence of corneal haze that hinders visualization of the angle structures during operation [14].

Filtering surgery in pediatric patients has been associated with lower success rates when compared to adult populations due to rapid and aggressive healing in this age group. For this reason, MMC is commonly used to augment trabeculectomy in this population. The use of MMC in pediatric glaucoma surgery is associated with numerous short- and long-term complications, and this mandates lifelong follow-up of these children [15, 16].

In the present study, trabeculectomy was performed as the primary surgical procedure to treat PCG since corneal clarity was not sufficient enough to permit angle surgery. The patients' mean age in this study was 2.5 ± 0.5 months, and in this very young age group, significant corneal cloudiness is a main presenting symptom that drives parents to seek medical care and may also represent the more aggressive prototype of developmental glaucoma, which is known to be less responsive to angle surgeries. Furthermore, tissue handling in this group represents a greater surgical challenge with the very thin, elastic sclera with low rigidity and the greater difficulty locating and inserting the trabeculotome into the canal of Schlemm.

Twenty five infants with bilateral PCG were enrolled in this study. One eye of each patient was assigned to receive a modified subscleral trabeculectomy (group I), and the other eye was assigned to receive MMC-augmented subscleral trabeculectomy (group II). Trimming of the 3 edges of the scleral bed just before conjunctival suturing at the end of the trabeculectomy is an added step by which the partial thickness scleral flap would be at a higher level than the edges of the bed, hence decreasing the resistance to aqueous outflow and enhancing free fluid egress from the AC. This modified surgical technique was adopted to maintain aqueous outflow without the use of MMC in order to avoid its complications. By this technique, enhancement of the survival of trabeculectomy is done via creation of three pockets for free fluid exit which is proposed to retard healing by space separation between the flap and the scleral bed edges especially in the early postoperative period until the bleb is established, and then the aqueous constituents maintain it after that.

There was no detected significant difference between group I and group II with regard to the patients' demographic data, preoperative mean corneal diameter axial length, or CD ratio (*P* > 0.05). There was a high statistical significant difference with regard to postoperative IOP values from preoperative values in both groups, but on subsequent follow-up, the differences in the mean IOP values were statistically insignificant between the two groups (*P* > 0.05).

Regarding the occurrence of postoperative complications, there were no statistical significant difference among both groups, as operative hyphema occurred in one eye (4%) in group I and 2 eyes (8%) in group II and early postoperative shallow AC was encountered in 3 eyes (12%) in group I and in 2 eyes (8%) in group II. Choroidal effusion developed in one eye (4%) in each group. Late postoperative drawn-up pupil occurred only in one eye (4%) in group I. High IOP with glaucoma topical medications was encountered in 4 eyes (16%) in group I and in 3 eyes (12%) in group II, which were considered as failure of treatment to control the high IOP because they necessitated further glaucoma surgical interventions.

The sclera in PCG eyes is under stretch that it usually gaps spontaneously once the side incisions are performed during creation of the scleral flaps, potentially and spontaneously producing the effect we intend to create surgically. The rationale for trimming the edges of the scleral bed was to enhance the potential space between the flap edges and the scleral bed, allowing free flow of aqueous and delaying the healing by washing the wound healing precursors. This was clinically supported by the results of this study which shows that the postoperative IOP behaved similarly in both groups.

## 5. Conclusion

Trimming of the scleral bed edges is a surgical step that can be added to the procedure to effectively control the IOP in infants with PCG, sparing the adjunctive use of MMC in this age group.

## Figures and Tables

**Table 1:** The patients' preoperative and demographic data in both groups.

	Group I (25 eyes)	Group II (25 eyes)	*P* value
Number of patients	25 patients with bilateral PCG		
Age	The mean age was 2.5 ± 0.5 months		
Gender	15 patients were males and 10 were females		
Laterality			
Right eye	11 eyes	14 eyes	0.53
Left eye	14 eyes	11 eyes	0.53
Preoperative IOP (mean ± SD) (mmHg)	31.6 ± 4.9	32.1 ± 4.0	0.69
Preoperative horizontal corneal diameter (mean ± SD) (mm)	12.5 ± 1.5	13 ± 1.2	0.199
Preoperative CD ratio (mean ± SD) (mm)	55.9 ± 1.8	57.5 ± 2.1	0.32
Preoperative axial length (mean ± SD) (mm)	20.3 ± 1.7	20.5 ± 1.1	0.38

**Table 2:** Absolute and qualified success in eyes of both groups.

	Group I (*N* = 25 eyes)		Group II (*N* = 25 eyes)		*X* ^2^	*P* value
	No	(%)	No	(%)		
Absolute success	19	76	17	68	0.397	0.53
Qualified success	2	8	5	20	1.5	0.22
Total success	21	84	22	88	0.166	0.68

**Table 3:** Complications recorded in the study groups.

	Group I (25 eyes)		Group II (25 eyes)		*P* value
	No	(%)	No	(%)	
Operative					
Hyphema	1	4	2	8	0.55
Early postoperative					
Shallow AC	3	12	2	8	0.64
Choroidal effusion	1	4	1	4	—
Late postoperative					
Drawn-up pupil	1	4	—	0.0	0.69
High IOP > 21 with medical treatment	4	16	3	12	0.38

**Table 4:** Comparison of bleb morphology between the 2 study groups using the IBAGS.

	Group I	Group II	*P* value
Bleb height	2.88 (2.05–3.11)	2.73 (2.23–2.99)	0.12
Bleb extent	2.44 (2.23–2.65)	2.54 (2.43–2.85)	0.23
Bleb vascularity	1.85 (1.82–2.29)	1.99 (1.72–2.49)	0.19
Bleb leak	0 (0–0)	0 (0–0)	—

## Data Availability

Data underlying the findings of this study are available from the corresponding author upon request.
